# Comparative physiological and transcriptomic analyses provide integrated insight into osmotic, cold, and salt stress tolerance mechanisms in banana

**DOI:** 10.1038/srep43007

**Published:** 2017-02-22

**Authors:** Wei Hu, Zehong Ding, Weiwei Tie, Yan Yan, Yang Liu, Chunlai Wu, Juhua Liu, Jiashui Wang, Ming Peng, Biyu Xu, Zhiqiang Jin

**Affiliations:** 1 Key Laboratory of Biology and Genetic Resources of Tropical Crops, Institute of Tropical Bioscience and Biotechnology, Chinese Academy of Tropical Agricultural Sciences, Xueyuan Road 4, Haikou, Hainan province, 571101, China; 2 Hainan Key Laboratory of Banana Genetic Improvement, Haikou Experimental Station, Chinese Academy of Tropical Agricultural Sciences, Yilong W Road 2, Haikou, Hainan Province, 570102, China

## Abstract

The growth, development, and production of banana plants are constrained by multiple abiotic stressors. However, it remains elusive for the tolerance mechanisms of banana responding to multiple abiotic stresses. In this study, we found that Fen Jiao (FJ) was more tolerant to osmotic, cold, and salt stresses than BaXi Jiao (BX) by phenotypic and physiological analyses. Comparative transcriptomic analyses highlighted stress tolerance genes that either specifically regulated in FJ or changed more than twofold in FJ relative to BX after treatments. In total, 933, 1644, and 133 stress tolerance genes were identified after osmotic, cold, and salt treatments, respectively. Further integrated analyses found that 30 tolerance genes, including transcription factor, heat shock protein, and E3 ubiquitin protein ligase, could be commonly regulated by osmotic, cold, and salt stresses. Finally, ABA and ROS signaling networks were found to be more active in FJ than in BX under osmotic, cold, and salt treatments, which may contribute to the strong stress tolerances of FJ. Together, this study provides new insights into the tolerance mechanism of banana responding to multiple stresses, thus leading to potential applications in the genetic improvement of multiple abiotic stress tolerances in banana.

In field conditions, plants usually have to confront multiple environmental stresses, such as extreme temperature, salinity, and drought. Plants have evolved a series of complicated mechanisms to confront these stressors so that they can survive and complete their life cycle^1^,^2^,^3^. The primary responses of plants to abiotic stress involve the perception and transduction of stress signals, activation of stress-associated genes and proteins, and ultimately resulting in metabolic and physiological changes^1^. Thus, the response of plants to environmental stress is an extremely complicated process. Previously, many studies has focused on the mechanisms of plants responding to a single environmental factor^1^,^4^,^5^,^6^,^7^,^8^,^9^,^10^,^11^. Although these studies have potential applications in crop improvement for environmental stress tolerance, the interactive improvement of multiple abiotic stress tolerances is a challenge. Therefore, there is a need to investigate the metabolic pathways and regulatory networks of multiple abiotic stress acclimations in plants and obtain candidate genes for manipulation to improve stress tolerance.

Banana (*Musa acuminata* L.) is a large monocotyledonous herbaceous plant that is widely distributed throughout tropical and subtropical countries. Banana is not only the most popular fruit but also one of the largest fruit crops, which is vital for food security for millions of people around the world^12^,^13^. Compared with some other crops, banana research has developed slowly, because banana is only planted as food for the largely impoverished continent of Africa^12^. Banana plants are extremely sensitive to water stress induced by drought, osmotic, salt, cold, and other environmental stressors because they have rapid growth rate, a permanent green canopy, and shallow roots^14^. The investigation of banana gene expression patterns in response to environmental stressors will pave the way for further understanding the regulatory networks of abiotic stress acclimation in plants and help to select candidate genes for manipulation to improve abiotic stress tolerances. Previous studies have identified some abiotic stress responsive genes at transcriptional levels in banana. Davey *et al*.^15^ used microarrays to show that 2910 banana genes had differential expression levels after drought treatment. Lee *et al*.^16^ reported that more than 2980 expressed unigenes showed transcriptional changes when banana roots were subjected to salt stress based on RNA-seq. As reported by Yang *et al*.^2^, expression of 10 and 68 genes changed after 3 and 6 h of cold treatment respectively in plantain, whereas expression of 40 and 238 genes changed in banana using RNA-seq. Recently, 92 genes were commonly identified as differentially expressed in the three genotypes of banana after osmotic treatment^17^. However, no evidence has been found to integratedly investigate the stress tolerance mechanisms of banana to multiple environmental stressors, including osmotic, cold, and salt stresses.

Different varieties of the same species can also exhibit a high degree of genetic variability for abiotic stress tolerance. In particular, the “ABB” banana genotypes are more tolerant to drought and other abiotic stresses than other genotypes^18^. Thus, the banana varieties based on “ABB” genotype can be used as a crucial genetic resource for crop improvement for abiotic stress. In the present study, we aimed to integratedly study the osmotic, cold and salt stress tolerance mechanisms of banana by comparative physiological and transcriptomic analyses of the BaXi Jiao (AAA genotype) and Fen Jiao (ABB genotype).

## Methods

### Plant materials and treatments

BaXi Jiao (*Musa acuminate* L. AAA group cv. Cavendish, BX) is a high quality fruit, but it is sensitive to abiotic stress. Fen Jiao (*Musa* ABB Pisang Awak, FJ) has the characteristics of good flavor and strong tolerance to abiotic stress^19^,^20^. Young banana seedlings of BX and FJ at the five-leaf state were acquired from the banana tissue culture center (Institute of Banana and Plantain, Chinese Academy of Tropical Agricultural Sciences, Danzhou). Banana seedlings of BX and FJ grew in soil under a growth chamber (16 h light/8 h dark cycle; 200 μmol · m^−2^ · s^−1^ light intensity; 28 °C; 70% relative humidity). For osmotic, cold, and salt treatment, banana seedlings were treated with 200 mM mannitol for 18 d, 4 °C for 48 h following 5 d recovery, and 300 mM NaCl for 32 d, respectively. For physiological analyses, leaves were sampled from 0d, 7d, 10d, 15d, and 18d osmotic treatments, from 0 h, 10 h, 22 h, 48 h cold, and 5 d recovery treatments, and from 0d, 7d, 12d, 24d, and 32d salt treatments. Three biological experiments were performed for each sample.

### Physiological analyses

Relative water content was tested based on Barrs and Weatherley^21^. Fresh banana leaves were weighed as the Fresh weight (FW). Then the leaves were soaked in distilled water for 4 h and weighed as the turgid weight (TW). After that, banana leaves were dried at 80 °C for 24 h and the leaves were weighed as dry weight (DW). Relative water content was calculated using the equation: RWC (%) = [(FW - DW)/(TW - DW)] × 100. Ion leakage was examined with the methods described by Xu *et al*.^22^. The osmotic potential was determined with a dewpoint PotentiaMeter based on the manufacturer’s instruction (WP4C, DECAGON, USA).

### Transcriptomic analyses

Banana leaves were collected from control and osmotically treated plants for 7 d, salt treated plants for 7 d, and cold treated plants for 22 h. The second young leaf of each plant was detached from the top of six independent plants at each time point for each biological experiment. Leaves from the six plants were cut into pieces and mixed well. Two biological experiments were performed for each sample. Total RNA was extracted from banana leaves of BX and FJ using a RNA extraction kit (DP432, TIANGEN, Beijing, China). A total of 3 μg total RNA from each sample was converted into cDNA using a RevertAid First-Strand cDNA Synthesis Kit (Promega, Madison, WI, USA). Sixteen cDNA libraries were constructed and were subsequently sequenced with an Illumina GAII following the protocol, which resulted in 1334.1 million 90-bp raw reads. The raw data generated from the 16 libraries was shown in Table S1 and was deposited in NCBI-SRA database (accession number: PRJNA343716). The sequencing depth was 5.36X on average. With the help of FASTX-toolkit, adapter sequences in the raw reads were removed, which produced 761.5 million clean reads used for further analysis. Sequence quality was assessed with FastQC. On average, 64.3% clean reads were mapped to DH-Pahang (*Musa acuminate*, A-genome, 2n = 22) genome using Tophat v.2.0.10^23^,^24^. Cufflinks with alignment files were used to perform the transcriptomic assemblies^25^. Reads Per Kilo bases per Million reads (RPKM) was employed to calculate gene expression levels, and RPKM of two replicates was averaged. Differentially expressed (DE) genes were determined by DEGseq based on the read count of two replicates for each gene (fold change ≥ 2; P-value ≤ 0.001)^26^.

### qRT-PCR analyses

To confirm the transcriptional levels of several genes identified by transcriptomic analyses under osmotic, cold, and salt treatments, qRT-PCR was carried out using Mx3000 P (Stratagene, CA, USA). The optimal concentrations of templates and primers were determined by a series of primer and template dilutions. The specificity and efficiency of primer pairs were examined using agarose gel electrophoresis, melting curve, and sequencing analyses. Amplification efficiencies of primer pairs were in the range 0.92–1.07. The chosen primer pairs are listed in Table S2. Internal controls (*MaRPS2* and *MaUBQ2*) were employed to normalize the expression levels of target genes^27^. The relative expression levels of the target genes were calculated by 2^−ΔΔCt^ method^28^.

## Results

### Performance and physiological response of BX and FJ under osmotic, salt, and cold treatments

**Figure 1:**
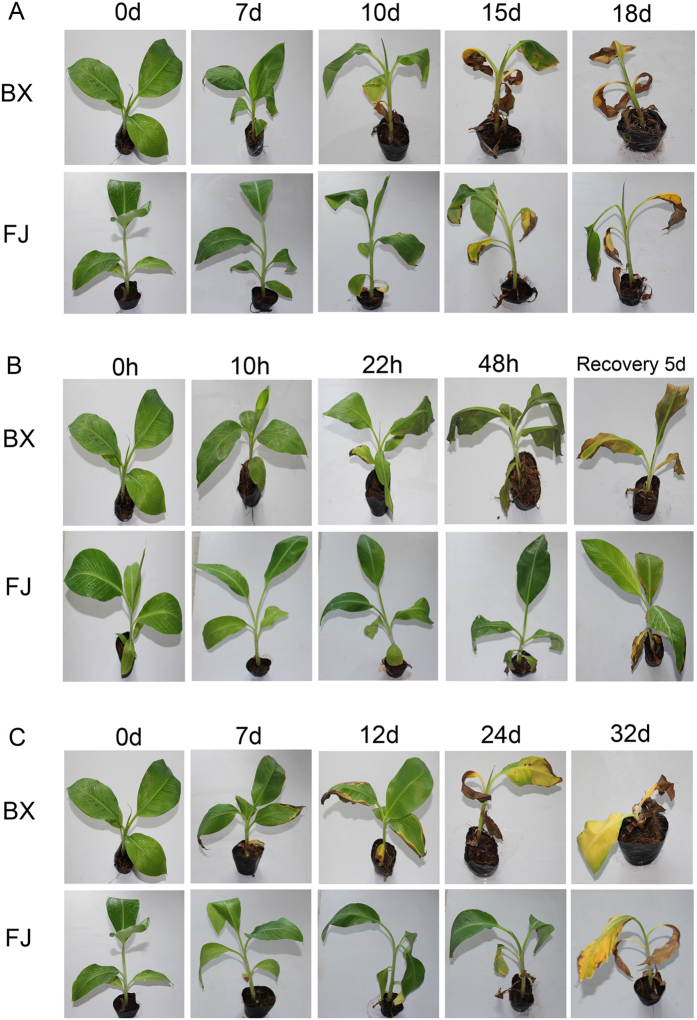
Phenotypic analyses of BX and FJ in response to osmotic (**A**), cold (**B**), and salt (**C**) stresses. Five leaf stage banana seedlings were subjected to 200 mM mannitol, cold (4 °C), and 300 mM NaCl treatments and photos were taken at different time points.

To accurately evaluate the tolerance of osmotic, salt, and cold stresses between BX and FJ, young banana seedlings of BX and FJ with uniform growth during the five- leaf stage were treated with various stresses (Fig. 1). Under osmotic treatment, the third, fourth, and fifth BX leaves from the top obviously wilted and drooped after 7 days treatment, whereas the symptom was much weaker in FJ leaves (Fig. 1A). Under cold treatment, all BX leaves significantly drooped and the edge of the fifth leaf became necrosis after 22 h treatment, whereas only the third, fourth, and fifth leaves of FJ drooped slightly (Fig. 1B). Under 7 d of salt treatment, the edges of second, third and fourth BX leaves exhibited chlorosis and the fifth leaves of BX displayed necrosis, while only the fifth leaves of FJ plants showed chlorosis (Fig. 1C). The chlorosis and necrosis symptoms in BX were more severe than that in FJ during 10–18 d osmotic, 48 h cold and 5 d recovery, and 12–32 d salt treatments (Fig. 1). These results indicated that as compared with BX, FJ was more tolerant to osmotic, cold, and salt stresses.

**Figure 2:**
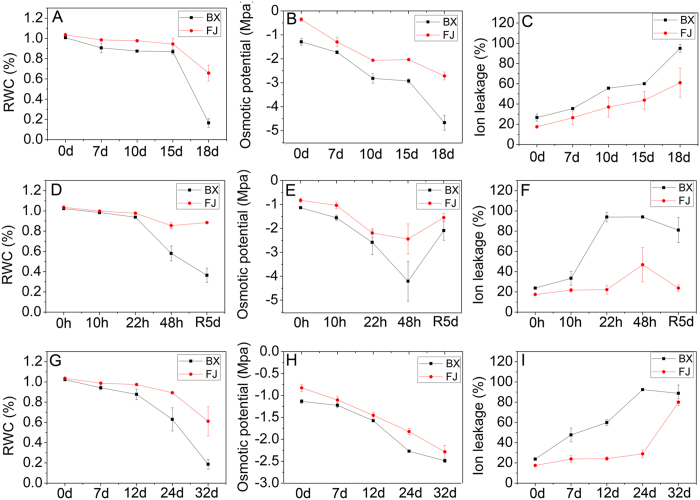
Physiological analyses of BX and FJ in response to osmotic (**A–C**), cold (**D–F**), and salt (**G–I**) stresses. Relative water content, osmotic potential, and ion leakage were examined in BX and FJ under normal and treated conditions. Data are means ± SD calculated from three independent experiments.

To further confirm the tolerances to osmotic, cold, and salt stresses between BX and FJ, several physiological indices were measured (Fig. 2). During osmotic, cold, and salt treatments, both BX and FJ showed decreased relative water content and osmotic potential and increased ion leakage. After cold treatment following recovery for 5 d, the osmotic potential increased and the ion leakage decreased both in BX and FJ. This suggested that these stressors led to the injury of banana leaves. Notably, the relative water content and osmotic potential were higher, and the ion leakage was lower in FJ than in BX after treatments, further confirming the strong tolerances of FJ compared with BX.

### Identification of osmotic, salt, and cold stress tolerance genes

To get a better understanding of the mechanism underlying the stress tolerance, comparative transcriptome analysis was performed between BX and FJ respectively under 7 d osmotic, 22 h cold, and 7 d salt treatments, because slight stress symptoms began to appear at these time points in banana seedling leaves. Overall, the replication of samples looks good, as the replications were all closely clustered (Fig. S1). Since FJ is more tolerant than BX under osmotic, cold, and salt treatments, we pay more attention to the genes that either specifically regulated in FJ or changed more (e.g., ≥ twofold) in FJ relative to BX. These two series of genes are probably involved in stress tolerance of banana, thus defined as stress tolerance genes in FJ relative to BX.

**Figure 3:**
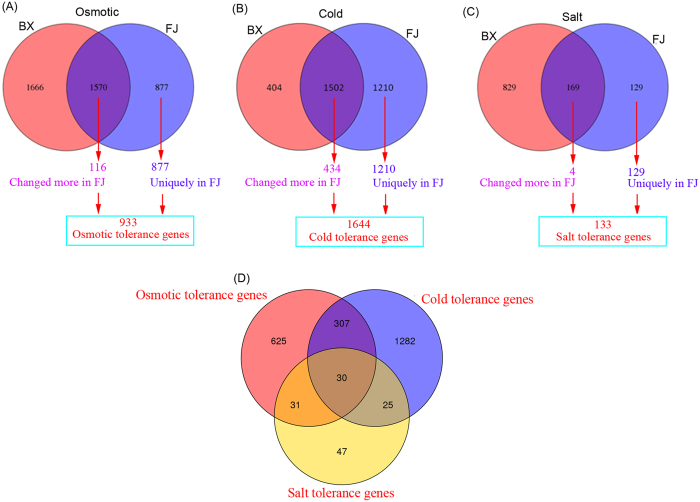
Distribution of differentially expressed genes (fold change ≥ 2; *P*-value ≤ 0.001) in BX and FJ after osmotic, salt and cold treatments. Venn diagram showing the differentially expressed genes in BX and FJ after osmotic (**A**), cold (**B**), and salt (**C**) treatments. (**D**) Venn diagram showing the osmotic, cold, and salt tolerance genes in FJ relative to BX. The tolerance genes were defined as either specifically regulated in FJ or changed more (e.g., ≥ twofold) in FJ relative to BX.

After 7 d of osmotic treatment, 3236 and 2447 DE genes were identified from BX and FJ, respectively (Table S3). Among these, 1666 genes were exclusively identified in BX; 877 genes were uniquely found in FJ; and 1570 genes were commonly regulated in both BX and FJ (Fig. 3A). Among the commonly up- or down-regulated genes, the expression of 116 genes was changed more in FJ than in BX. Thus, a total of 993 genes were identified as osmotic tolerance genes in FJ relative to BX (Fig. 3A; Table S4). GO enrichment analyses showed that the majority of osmotic tolerance genes belonged to 84 categories, including oxidoreductase activity, defense response, response to stress, response to stimulus, abscisic acid-activated signaling pathway, response to reactive oxygen species, and response to oxidative stress, etc (Table S5).

After 22 h of cold treatment, 1906 and 2712 DE genes were identified from BX and FJ, respectively (Table S6). Among these, 404 genes were exclusively identified in BX; 1210 genes were uniquely found in FJ; and 1502 genes were commonly regulated by cold stress in both BX and FJ (Fig. 3B). Among the commonly up- or down-regulated genes, the expression of 434 genes was changed more in FJ than in BX. Thus, a total of 1644 genes were identified as cold tolerance genes in FJ relative to BX (Fig. 3B; Table S7). GO enrichment analyses showed that these cold tolerance genes belonged to 110 categories, including abscisic acid binding, oxidoreductase activity, defense response, response to abiotic stimulus, and response to stress, etc (Table S8).

After 7 days of salt treatment, 998 and 298 DE genes were identified from BX and FJ, respectively (Table S9). Among these, 829 genes were exclusively identified in BX; 129 genes were uniquely found in FJ; and 169 genes were commonly regulated by salt stress in both BX and FJ (Fig. 3C). Among the commonly up- or down-regulated genes, the expression of only 4 genes was changed more in FJ than in BX. Thus, a total of 133 genes were identified as salt tolerance genes in FJ relative to BX (Fig. 3C; Table S10). GO enrichment analyses showed that these salt tolerance genes belonged to 28 categories, including response to reactive oxygen species, response to hydrogen peroxide, response to abiotic stimulus, response to oxidative stress, response to stress, and abscisic acid metabolic process, etc (Table S11).

Together, these results indicated that genes involved in abscisic acid and ROS pathways were commonly regulated by osmotic, cold, and salt treatments in banana.

### Identification of the tolerance genes commonly regulated by osmotic, cold, and salt stress

To integratedly investigate the drought, cold and salt stress tolerant mechanisms of banana, we performed integrated analysis of the stress tolerance genes in FJ relative to BX. A total of 30 tolerance genes were found to be commonly regulated by osmotic, cold, and salt stress in FJ relative to BX (Table 1). Among these, 22 genes were more up-regulated (at least twofold) in FJ than in BX. Interestingly, these genes included 6 transcription factors (3 ERF, 2 ZFP, and 1 WRKY), 3 heat shock protein, and 1 E3 ubiquitin-protein ligase, etc. In contrast, 8 genes were more down-regulated (at least twofold) in FJ than in BX, including glutamate dehydrogenase, vacuolar amino acid transporter, calcium uniporter, etc (Table 1). These genes may be the key regulators commonly involved in multiple stresses (e.g., osmotic, cold, and salt) tolerance in banana.

### Osmotic, cold, and salt tolerance genes involved in ABA-dependent and ABA-independent signaling network

**Figure 4:**
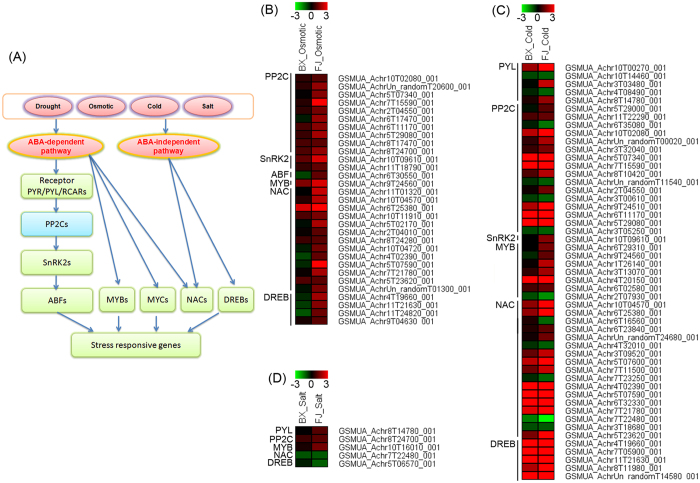
Expression patterns of osmotic, cold, and salt tolerance genes involved in ABA-dependent and ABA-independent signaling network. (**A**) Generalized model of the ABA-dependent and ABA-independent signaling network. Osmotic (**B**), cold (**C**), and salt (**D**) tolerance genes associated with ABA signaling network were identified from the DE genes. Log2 based RPKM value was used to create the heat map. The scale represents the relative signal intensity of RPKM values. PYL, Abscisic acid receptor; PP2C, Protein phosphatase 2 C; SnRK2, Osmotic stress/abscisic acid-activated protein kinase; ABF, ABRE binding factors; MYB, Myb transcription factor; MYC, Myc transcription factor; NAC, Nac transcription factor; DREB, Dehydration-responsive element-binding protein.

Previously, ABA-dependent and ABA-independent signaling network have been clarified as shown in Fig. 4A 29. To gain a deep insight into the ABA-mediated tolerance mechanisms of FJ relative to BX, the osmotic, cold, and salt tolerance genes related to ABA-dependent and ABA-independent signaling network were identified from the transcriptomic data (Fig. 4; Table S12).

Under osmotic treatment, a total of 31 osmotic tolerance genes in FJ relative to BX were participated in ABA-dependent and ABA-independent signaling network. These genes belonged to PP2C, SnRK2, ABF, MYB, NAC, and DREB families. Notably, all these genes were more upregulated in FJ than in BX (Fig. 4B).

Under cold treatment, a total of 51 cold tolerance genes in FJ relative to BX were involved in ABA-dependent and ABA-independent signaling network. These genes belonged to PYL, PP2C, SnRK2, MYB, NAC, and DREB families. Among them, 39 genes were more upregulated in FJ than in BX, whereas 12 genes were more downregulated in FJ relative to BX (Fig. 4C).

Under salt treatment, 5 salt tolerant genes in FJ relative to BX were participated in ABA-dependent and ABA-independent signaling network. Among them, PYL, PP2C, and MYB were more upregulated in FJ than in BX, whereas NAC and DREB were more downregulated in FJ relative to BX (Fig. 4D).

### Osmotic, cold, and salt tolerance genes related to ROS signaling network

**Figure 5:**
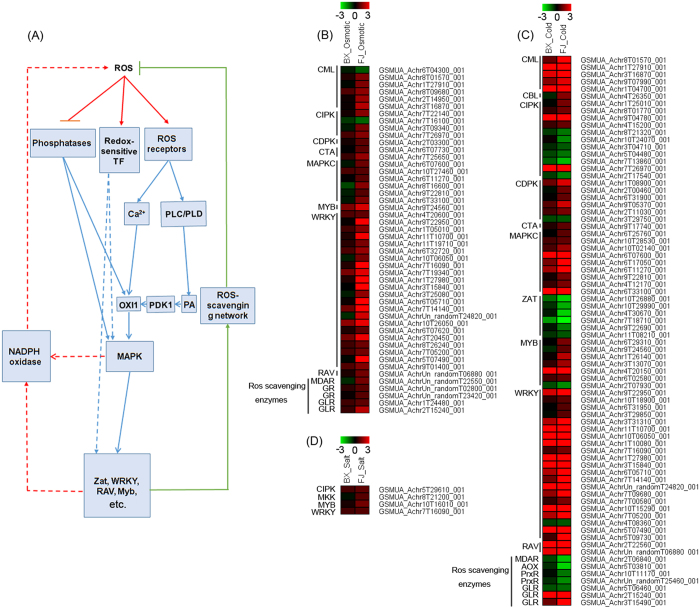
Expression patterns of osmotic, cold, and salt tolerance genes involved in ROS signal network. (**A**) Generalized model of the ROS signal transduction pathway. Osmotic (**B**), cold (**C**), and salt (**D**) tolerance genes associated with ROS signaling network were identified from the DE genes. Log2 based RPKM value was used to create the heat map. The scale represents the relative signal intensity of RPKM values. CML, Calcium-binding protein; CBL, Calcineurin B-like protein; CIPK, CBL-interacting protein kinase; CDPK, Calcium-dependent protein kinase; CTA, Calcium-transporting ATPase; MPKC, Mitogen-activated protein kinase cascade; ZAT, Zinc finger protein; MYB, Myb transcription factor; WRKY, Wrky transcription factor; RAV, AP2/ERF and B3 domain-containing transcription repressor; MDAR, Monodehydroascorbate reductase; GR, Glutathione reductase; GLR, Glutaredoxin; AOX, Alternative oxidase; PrxR, Peroxiredoxin.

Previously, ROS signaling network has been clarified as shown in Fig. 5A 30,31. To better understand the ROS-mediated tolerance mechanisms of FJ relative to BX, the osmotic, cold, and salt tolerance genes related to ROS signaling network were identified from the transcriptomic data (Fig. 5; Table S13).

Under osmotic treatment, 48 osmotic tolerance genes in FJ relative to BX is involved in ROS signaling network. These genes belonged to CML, CIPK, CDPK, CTA, MAPKC, MYB, WRKY, and ROS scavenging enzymes. Notably, all these genes, except for CML GSMUA_Achr6T04300_001and CIPKGSMUA_Achr7T16100_001, were more upregulated in FJ relative to BX (Fig. 5B).

Under cold treatment, a total of 76 cold tolerance genes in FJ relative to BX is involved in ROS signaling network. Among them, 56 genes were more upregulated in FJ than in BX. These mainly distributed in CML, CBL, CDPK, CTA, MAPKC, MYB, WRKY, and RAV families. In contrast, 20 genes were more downregulated in FJ relative to BX. These mainly belonged to CIPK, ZFP, and ROS scavenging enzymes (Fig. 5C).

Under salt treatment, 4 salt tolerance genes, including CIPK, MKK, MYB, and WRKY, is involved in ROS signaling network. All these genes were more upregulated in FJ relative to BX (Fig. 5D).

### Validation of the DE genes by qRT-PCR

**Figure 6:**
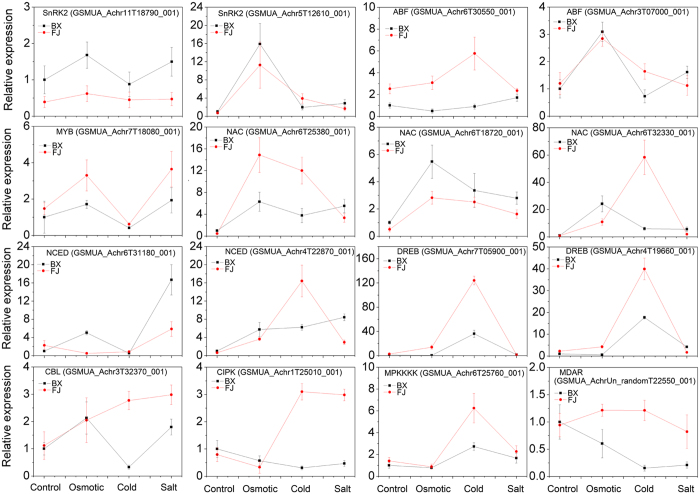
Relative expression levels of 16 genes after osmotic, salt and cold treatmetns in BX and FJ were examined by qRT-PCR. The mRNA fold difference was relative to that of untreated samples of BX used as calibrator. Data are means ± SD of n = 3 independent experiments.

To validate the transcriptomic data, expression levels of 16 DE genes in BX and FJ were tested under osmotic, cold and salt treatments by using qRT-PCR. These genes include SnRK2, ABF, MYB, NAC, NCED, and DREB related to ABA-dependent and ABA-independent signaling network; and CBL, CIPK, MAPKKK, and MDAR associated with ROS signal network. As shown in Fig. 6 and Fig. S2, most of the selected genes, except for DREB (GSMUA_Achr7T05900_001 and GSMUA_Achr4T19660_001), NCED (GSMUA_Achr6T31180_001), CIPK (GSMUA_Achr1T25010_001), and MAPKKK (GSMUA_Achr6T25760_001) in several points, showed similar trends between qRT-PCR data and RNA-seq data. The reasons for discrepancy between expression analysis by transcriptome and qRT-PCR might due to that genes have different alternative forms: RNA-seq could capture the expression (mapped reads) of all alternative forms for a gene, while qRT-PCR might capture the expression of only one alternative form^32^. Secondly, RNA-seq and qRT-PCR should exhibit consistent result for genes with high expression and sufficient significance, but this may not true for genes with low expression and subtle difference^33^. Moreover, the sensitivity of these two technologies is also different.

## Discussion

FJ (ABB genotype) has strong tolerance to abiotic stress, including osmotic, cold, and salt^19^,^20^. In contrast, BX (AAA genotype) is sensitive to abiotic stress relative to FJ^19^,^20^. In the present study, we confirmed that FJ is more tolerant to osmotic, cold, and salt stresses than BX based on the phenotypic and physiological analyses (Figs 1 and 2), which is consistent with previous studies. Thus, comparative transcriptomic analyses of these two banana varieties could uncover stress tolerance mechanisms and identify stress tolerance genes of banana.

### Candidates for improving multiple stress tolerance of banana

Integrated investigation of the transcriptional response of banana to abiotic stress, including drought/osmotic, salt, and cold stresses is beneficial for understanding the regulatory networks of multiple abiotic stress acclimations and obtaining candidate genes to improve stress tolerance. In this study, we identified 933, 1644, and 133 tolerance genes in FJ relative to BX after osmotic, cold, and salt treatments, respectively (Fig. 3). Further integrated analyses found that 30 tolerance genes could commonly regulated by osmotic, cold, and salt stress in FJ relative to BX (Table 1). Interestingly, 6 genes encoding transcription factors (3 ERF, 2 ZFP, and 1 WRKY) were more upregulated in FJ than in BX. Many ERF genes from various species have been confirmed to positively regulate plants tolerance to abiotic stress, such as, OsEREBP1, PsAP2, RAP2^34^,^35^,^36^. Besides, ZFP and WRKY were also reported to play a positive role in plants’ tolerance to abiotic stress. For example, salt, osmotic, and ABA treatments resulted in increased transcripts of IbZFP1 and IbZFP1 overexpression improved Arabidopsis tolerances to salt and drought stresses^37^. Banana *MusaWRKY71* was upregulated by various abiotic stress stimuli, and overexpression of *MusaWRKY71* in banana could improve banana tolerance to oxidative and salt stresses^10^. These evidences revealed the crucial roles of these transcription factors in improving plants’ tolerance to abiotic stress. Thus, FJ might have a more efficient transcriptionally regulatory mechanism mediated by transcription factors, which contributes to their strong tolerance to abiotic stress.

Heat shock protein (HSP) plays a role as molecular chaperone in protecting plants against abiotic stresses. Accumulating evidences have suggested the roles of HSPs in plants responding to abiotic stress^38^. For example, *Rosa chinensis RcHSP17.8* transcripts increased after various abiotic stress treatments. Overexpression of *RcHSP17.8* enhanced tolerance to drought, osmotic, salt, and heat stresses^39^. Here, we found 3 HSPs were more upregulated in FJ than in BX, indicating the important role of HSPs in banana tolerant to abiotic stress.

In plants, numerous biological processes were regulated by ubiquitin-mediated posttranslational modification^40^. E3 ubiquitin ligase functions on recognizing protein substrate and catalyzing the transfer of ubiquitin from the E2 to the protein substrate. Some biochemical and genetic evidences also support the role of E3 ubiquitin-protein ligase in abiotic stress response^40^,^41^,^42^. This suggested that E3 ubiquitin-protein ligase-mediated posttranslational modification may be involved in abiotic stress tolerance of banana.

### The improved ABA-dependent and -independent signaling network contribute to the strong tolerance of FJ

Abscisic acid (ABA) plays a central role in plants’ response to abiotic stress by regulating the expression of numerous genes and related physiological process^29^,^43^,^44^,^45^. In recent years, ABA perception and signal transduction have been elucidated showing that RCAR/PYR/PYL ABA receptors, group A PP2Cs, and SnRK2s control the ABA signaling pathway in land plants. SnRK2s can activate AREB/ABFs by phosphorylation in the ABA-dependent signaling network induced by abiotic stress. Additionally, other transcription factors, such as MYB, MYC, and NAC were also reported to be involved in ABA signaling pathway when responding to abiotic stress. Besides, DREB and NAC transcription factors play an important role by regulating stress-responsive genes in ABA-independent pathway^29^,^45^. However, ABA-mediated stress tolerance mechanisms remains unclear in banana.

In the present study, the DE genes involved in ABA and stress related process were widely enriched by all three treatments according to GO enrichment analyses (Tables S5, S8 and S11). Further analyses identified 31, 51, and 5 genes involved in ABA-dependent and -independent signaling network as osmotic, cold, and salt tolerance genes in banana, respectively (Fig. 4). Under cold treatment, most of the genes in PYL, PP2C, SnRK2, MYB, NAC, and DREB families were more upregulated in FJ than in BX. Under osmotic treatment, all the genes in PP2C, SnRK2, ABF, MYB, NAC, and DREB families were more upregulated in FJ than in BX. Under salt treatment, PYL, PP2C, and SnRK2 genes were more upregulated in FJ than in BX. Accumulated evidences have suggested that most members of SnRK2, ABF, MYB, NAC and DREB families play a positive role in plants’ response to abiotic stresses^46^,^47^,^48^,^49^,^50^. Thus, the improved ABA-dependent and -independent signaling network in FJ may contribute to its strong tolerance to abiotic stress.

Previously, PP2Cs were demonstrated as negative factors of ABA signaling network based on biochemical and genetic evidence^51^. However, most PP2C members were induced at transcriptional levels under osmotic, cold, salt and drought treatments in Arabidopsis^52^. Thus, the function of PP2Cs is not correlated with their expression patterns in response to abiotic stress. In this study, we found that most of the PP2C members were more upregulated in FJ than in BX. This indicated that the activation of PP2C at transcriptional levels by abiotic stress was correlated with the robust tolerance of FJ. It is concluded that post-transcriptional regulatory mechanisms may be involved in PP2C-mediated ABA signaling transduction, which need to be further clarified by future studies.

### The improved ROS signaling network is involved in the strong tolerance of FJ

There is significant ROS accumulation under abiotic stress conditions, which causes oxidative damage and eventually resulting in cell death. Controlling ROS toxicity enables ROS such as H_2_O_2_ or O2^−^ to act as signaling molecules during evolution^30^. ROS signaling have been widely implicated in plants responding to abiotic stresses^53^. ROS have also been recognized as key players in the complex signaling network of plant responses to abiotic stress. The key components of ROS signaling network have been clarified in plants^30^. Plant cells sense ROS through at least three different mechanisms, including unidentified receptor proteins, redox-sensitive transcription factors, and direct inhibition of phosphatases. Downstream signaling events include calcium and phospholipid signaling pathways, and hence activate serine/threonine protein kinase (OXI1), MAPK cascades, NADPH oxidase, and transcription factors. However, ROS-mediated stress tolerance mechanisms remains unclear in banana.

Our GO analyses showed that ROS-related process was extensively enriched (Tables S5, S8 and S11). To address the question that how does banana perceive and transduce ROS signaling to improve stress tolerance, we identified 48, 76, and 4 genes related to ROS signaling network as osmotic, cold, and salt tolerance genes in banana, respectively (Fig. 5). Under cold treatment, most of genes in CML, CBL, CDPK, CTA, MAPKC, MYB, WRKY, and RAV families were more upregulated in FJ than in BX. Under osmotic treatment, most of genes in CML, CIPK, CDPK, CTA, MAPKC, MYB, WRKY, and ROS scavenging enzymes were more upregulated in FJ than in BX. Under salt treatment, all the 4 genes (CIPK, MKK, MYB, and WRKY) were more upregulated in FJ relative to BX. Previous studies have revealed that most of the members in CML, CPK, MPKC, MYB, WRKY and RAV families positively regulate plants tolerance to abiotic stress^48^,^54^,^55^,^56^,^57^,^58^,^59^. Thus, the ROS network was more active in FJ than in BX, which may contribute to the robust tolerance of FJ to abiotic stress.

When combating with abiotic stressors, plants have developed a complex enzymes-mediated antioxidant system to scavenge ROS and protect cells^1^. Many evidences have revealed that ROS scavenging enzymes play a positive role in plants tolerance to drought/osmotic stress^60^,^61^,^62^,^63^,^64^. In this study, we found that all the identified ROS scavenging enzymes were more upregulated in FJ than in BX after osmotic treatment. Thus, FJ uses a more efficient antioxidant system to maintain its strong tolerance to osmotic stress. However, this phenomenon was not observed under cold and salt treatments. Most of genes encoding ROS scavenging enzymes, except for GLRs, were more downregulated in FJ than BX after cold treatment. No genes encoding ROS scavenging enzymes were found to be differentially expressed between BX and FJ under salt treatment. This indicated that it is different for ROS signaling network-mediated stress tolerance mechanisms between osmotic and cold/salt response in banana. As is known, many components in ROS signaling network, such as CML, CDPK, MAPK, MYB, RAV, and WRKY, regulate plants tolerance to abiotic stress through multiple pathways^53^,^65^. It is possible that these genes function on increasing banana tolerance to cold and salt stresses through activating other pathways.

In summary, this study provides integrated insights into banana tolerance to multiple stressors, including drought, cold and salt stress. Firstly, we confirmed that FJ was more tolerant to multiple stressors than BX. Further comparative transcriptomic analyses identified some osmotic, cold, and salt tolerance genes in FJ. Secondly, we found that 30 tolerance genes could be commonly regulated by osmotic, cold, and salt stress in FJ relative to BX, indicating that these genes might confer banana tolerance to multiple stresses. Lastly, ABA and ROS signaling networks were found to be preferentially activated in FJ under osmotic, cold, and salt treatments, which may contribute to its strong tolerances. These findings could contribute to better understanding of the molecular basis of banana tolerance to multiple stresses, yield new insights into the multiple stresses tolerant mechanism of banana, and be helpful for genetic improvement of banana tolerances to multiple abiotic stress.

## Additional Information

**How to cite this article**: Hu, W. *et al*. Comparative physiological and transcriptomic analyses provide integrated insight into osmotic, cold, and salt stress tolerance mechanisms in banana. *Sci. Rep.* **7**, 43007; doi: 10.1038/srep43007 (2017).

**Publisher's note:** Springer Nature remains neutral with regard to jurisdictional claims in published maps and institutional affiliations.

## Supplementary Material

Supplementary Figures

Supplementary Dataset 1

## Figures and Tables

**Table 1:** Tolerance genes commonly regulated by osmotic, cold, and salt stress in FJ relative to BX.

Gene ID	BX_Osmotic	FJ_Osmotic	BX_Cold	FJ_Cold	BX_Salt	FJ_Salt	Functions
GSMUA_Achr7T06910_001	−0.963	2.948	4.522	7.040	0.829	1.705	Ethylene-responsive transcription factor
GSMUA_Achr6T10440_001	0.032	1.183	2.433	4.177	0.240	1.184	Ethylene-responsive transcription factor
GSMUA_Achr10T19030_001	0.557	2.123	−0.595	3.442	−0.773	1.144	Ethylene-responsive transcription factor
GSMUA_AchrUn_randomT04690_001	−0.347	3.896	3.536	7.154	0.620	1.672	RING-H2 zinc finger protein
GSMUA_Achr2T13410_001	0.143	2.443	3.913	5.112	0.912	1.181	Zinc finger protein
GSMUA_Achr7T16090_001	0.449	4.676	0.800	1.404	0.820	1.014	WRKY transcription factor
GSMUA_Achr10T11030_001	−0.561	2.666	−0.849	1.039	0.049	1.904	22.7 kDa class IV heat shock protein
GSMUA_Achr10T22810_001	−0.099	1.224	−0.992	1.061	0.348	1.417	Heat shock cognate 70 kDa protein
GSMUA_Achr10T22800_001	−0.514	1.104	−0.818	1.142	0.139	1.309	Heat shock cognate 70 kDa protein
GSMUA_Achr10T22580_001	0.632	3.288	3.197	5.958	0.991	1.582	E3 ubiquitin-protein ligase
GSMUA_AchrUn_randomT23790_001	0.119	4.766	−0.034	1.405	0.118	1.916	Naringenin, 2-oxoglutarate 3-dioxygenase
GSMUA_AchrUn_randomT00920_001	0.385	2.777	−0.311	1.131	0.831	1.695	Inner membrane protein
GSMUA_Achr2T11130_001	2.408	4.742	0.372	1.603	0.987	1.352	GDSL esterase/lipase
GSMUA_Achr9T29700_001	−0.082	3.163	1.755	4.971	0.572	1.338	CCR4-associated factor
GSMUA_Achr10T22990_001	0.607	1.459	0.926	1.376	0.608	1.182	Dolichyldiphosphatase
GSMUA_Achr10T00680_001	−0.561	2.625	4.517	7.597	−0.162	1.163	Nematode resistance protein-like
GSMUA_Achr5T22180_001	−0.321	4.161	4.921	8.083	0.521	1.157	Putative nuclease
GSMUA_Achr3T31780_001	1.346	3.421	0.576	2.000	−0.135	1.929	NA
GSMUA_Achr6T22540_001	0.205	2.748	0.579	2.728	−0.629	1.828	NA
GSMUA_Achr2T03690_001	1.039	2.920	−0.408	2.368	−0.170	1.524	NA
GSMUA_Achr9T22480_001	1.218	2.483	4.854	7.109	0.900	1.252	NA
GSMUA_Achr2T03700_001	−0.198	1.581	0.846	1.307	−0.288	1.076	NA
GSMUA_Achr2T12720_001	−0.597	−1.832	−0.323	−1.042	−0.424	−1.077	Glutamate dehydrogenase
GSMUA_Achr6T06880_001	−0.070	−3.745	0.288	−2.255	0.081	−1.973	Vacuolar amino acid transporter
GSMUA_Achr4T14630_001	−0.816	−4.114	−0.259	−1.872	−0.625	−2.549	Calcium uniporter protein
GSMUA_Achr5T18590_001	−0.241	−1.176	−0.072	−1.047	−0.450	−1.140	Ribulose bisphosphate carboxylase
GSMUA_Achr1T13130_001	0.725	−1.816	−0.095	−1.334	0.816	−1.251	Carboxyvinyl-carboxyphosphonate phosphorylmutase
GSMUA_Achr7T07610_001	−0.203	−2.822	−0.085	−2.782	−0.150	−1.345	Photosystem II reaction center W protein
GSMUA_Achr2T14680_001	−1.080	−2.947	−0.591	−2.917	−0.966	−1.273	NA
GSMUA_Achr8T14310_001	−0.644	−3.705	0.170	−1.829	−0.411	−1.945	NA

Log2 based RPKM value were shown.
