# Supplementary material for: Comparative physiological and transcriptomic analyses provide integrated insight into osmotic, cold, and salt stress tolerance mechanisms in banana
Source: Sci Rep. 2017 Feb 22;7:43007. doi: 10.1038/srep43007 (PMC5320444; doi:10.1038/srep43007)
Supplement: Supplementary Figures [file srep43007-s1.doc]

**Supplementary information for following article**

**Comparative physiological and transcriptomic analyses provide integrated insight into osmotic, cold, and salt stress tolerance mechanisms in banana**

Wei Hu^1#^*, Zehong Ding^1#^, Weiwei Tie^1^, Yan Yan^1^, Yang Liu^1^, Chunlai Wu^1^, Juhua Liu^1^, Jiashui Wang^2^, Ming Peng^1^, Biyu Xu^1^*, Zhiqiang Jin^1,2^*

^1^Key Laboratory of Biology and Genetic Resources of Tropical Crops, Institute of Tropical Bioscience and Biotechnology, Chinese Academy of Tropical Agricultural Sciences, Xueyuan Road 4, Haikou, Hainan province, 571101, China

^2^Hainan Key Laboratory of Banana Genetic Improvement, Haikou Experimental Station, Chinese Academy of Tropical Agricultural Sciences, Yilong W Road 2, Haikou, Hainan Province, 570102, China

* Corresponding author:

Wei Hu (huwei2010916@126.com)

Biyu Xu ([biyuxu@126.com](mailto:biyuxu@126.com));

Zhiqiang Jin ([18689846976@163.com](mailto:18689846976@163.com))

^#^These authors contributed equally to this work.


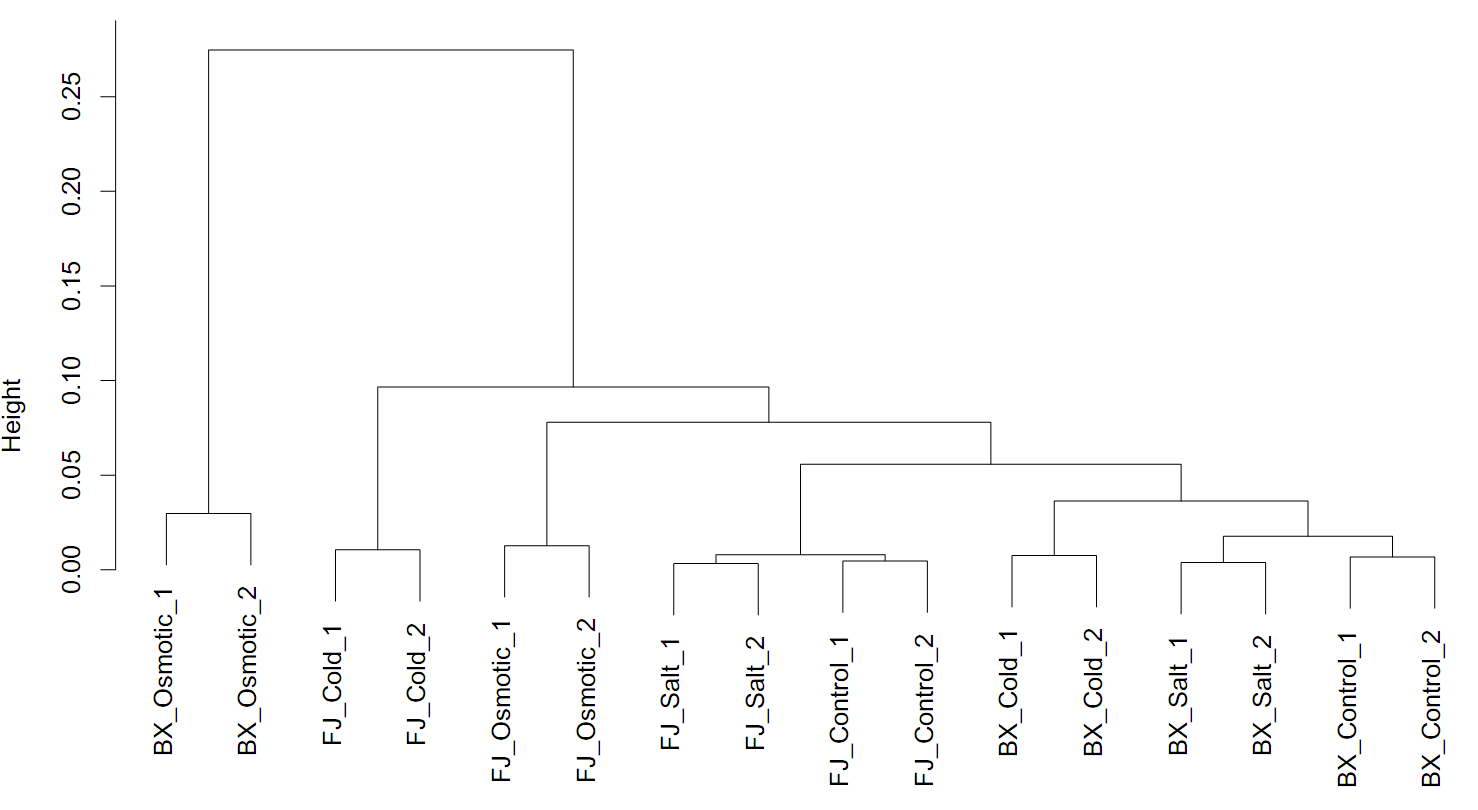


**Supplementary Figure S1. Clustering of samples used in this study.** Overall, the replication of samples looks good, as the replications were all closely clustered.

**
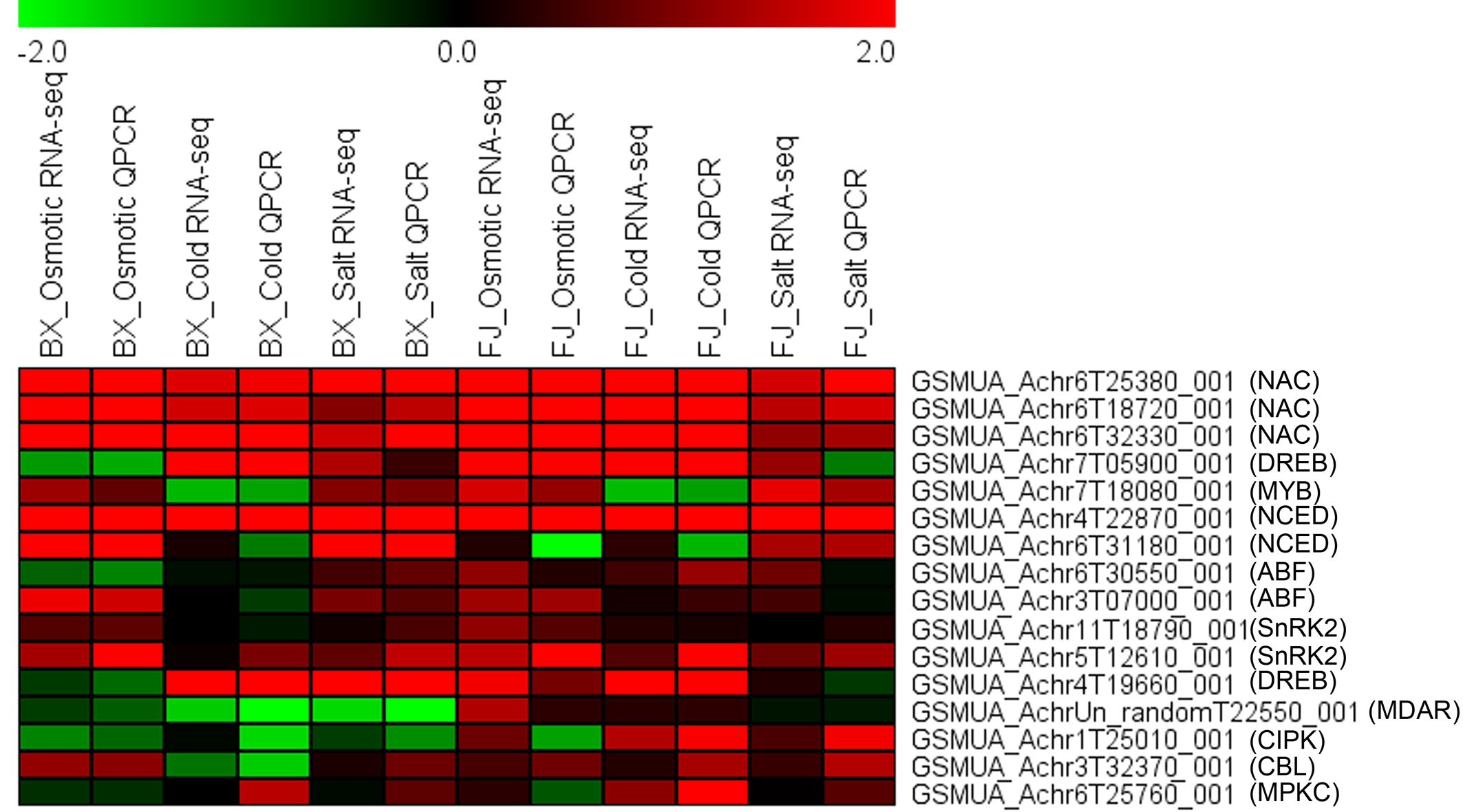
**

**Supplementary Figure S2. Comparison of the expression levels of the selected 16 genes after osmotic, salt and cold treatments by RNA-seq and QPCR.** Log2 based value was used to create the heat map. The scale represents the relative signal intensity.
